# Familial Testicular Germ Cell Tumor in Two Brothers With Emery Dreifuss Muscular Dystrophy Caused by an FHL-1 Mutation: A Case Report

**DOI:** 10.7759/cureus.38946

**Published:** 2023-05-12

**Authors:** Markus Angerer, Christian Wülfing, Klaus-Peter Dieckmann

**Affiliations:** 1 Department of Urology, Asklepios Klinik Altona, Hamburg, DEU

**Keywords:** genetic oncology, fhl-1 mutation, familial germ cell tumor, emery-dreifuss muscular dystrophy, testicular germ cell tumor

## Abstract

Testicular germ cell tumor (GCT) is a rare disease, accounting for no more than 1.5% of all neoplasms in males, but represents the most common tumors in adolescents and young men in Western countries. There is also consensus about the involvement of genetic factors in the etiology of testicular GCT. Familial occurrence of testicular GCT is observed in 1-2% of all cases with GCT.

We report the unique case of two brothers, both afflicted with inherited Emery-Dreifuss muscular dystrophy (EDMD) and both developing testicular GCT in young adulthood.

EDMD is a rare muscular dystrophy, characterized by the triad of joint contractures, slowly progressive muscle weakness, and cardiac involvement. EDMD is not a homogeneous clinical entity because it is associated with various gene mutations. One common mutation relates to the Four and a half Limb domain protein 1 (FHL-1) gene. To date, there have been no GCT cases linked with FHL-1 mutations and no malignant disease has been found associated with EDMD.

## Introduction

Testicular germ cell tumor (GCT) is a rare disease, accounting for no more than 1.5% of all neoplasms in males. However, GCTs represent the most common tumors in adolescents and young men in Western countries [1]. The etiology of testicular GCT is unknown, but there is solid evidence for an increased risk of GCT in cases of undescended testis and in patients with anteceding unilateral testicular GCT [2]. There is also consensus about the involvement of genetic factors in the etiology of testicular GCT, which is based on the much higher incidence of the disease in male family members of patients with GCT as compared to other males. Further evidence for the involvement of genetic factors comes from geographic-ethnic observations, with a significantly higher incidence of GCT in young males of Caucasian descent than in other ethnic groups [3]. Curiously, Maori men in New Zealand have a much higher incidence of GCT than males of European origin and of other ethnic groups in that country [4]. Finally, testicular GCT has been found to be associated with trisomy 21 (Down's syndrome) [5]. As there is no experimental or animal model of GCT, the entire knowledge of the disease's etiology is derived from epidemiological and clinical observations. Therefore, any unprecedented observation relating to this issue can be of value in terms of either supporting existing theories or generating novel hypotheses.

We report the novel cases of two brothers, both afflicted with inherited Emery-Dreifuss muscular dystrophy (EDMD) and both developing testicular GCT in young adulthood.

## Case presentation

A 29-year-old healthy man of Caucasian descent presented with a palpable, firm mass in the right testicle. Family history revealed a known genetic FHL-1 mutation, with the brother and mother of the patient being afflicted with the same mutation. Diagnosis of familial EDMD had been established by the summary of clinical features, muscle biopsy, and cardiac magnetic resonance imaging. The FHL-1 mutation was diagnosed by genetic analysis (microsatellite markers with linkage to locus Xq26-28).

Upon physical examination, the patient was overall in good condition, particularly with no disorders of the cardiovascular system. However, there were mild musculoskeletal symptoms, mainly an inability to extend both forearms. Scrotal sonography disclosed a strongly hypoechoic lesion sized 2.8 cm at the upper pole of the right testis.

Serum tumor markers were elevated: alpha-fetoprotein 41.5 ng/ml (reference limits (rl) <8.0 ng/ml), beta human chorionic gonadotropin (ßHCG) 4.97 mIU/ml (rl) <2 U/l), and lactate dehydrogenase 193 U/L (rl <250 U/L). After radical inguinal orchiectomy, all markers dropped into normal ranges.

Histological examination revealed a mixed non-seminomatous germ cell tumor (90% embryonal carcinoma, 10% yolk sac tumor) sized 3.2 cm, with vascular invasion and adjacent germ cell neoplasia in situ, corresponding to pathologic TNM classification, pT2 L0 V1 Pn0 R0. The radiological staging did not reveal metastases. Thus, the patient was classified as clinical stage 1b. He received one course of adjuvant cisplatin-etoposide-bleomcyin regimen (BEP) chemotherapy. The patient is well with no sign of recurrence six months after completion of treatment.

The older brother of the patient had been diagnosed with a right-sided non-seminomatous germ cell tumor at the age of 30 years. Histology involved a mixed non-seminomatous germ cell tumor (80% embryonal carcinoma, 13% yolk sac tumor, 5% chorionic carcinoma, and 2% teratoma), corresponding to the pathologic TNM-classification, pT2 NX L1 V0 Pn0 R0. Of note, a contralateral biopsy had disclosed germ cell neoplasia in situ. Radiological staging did not disclose any metastases, however, serum tumor markers did not return to the normal range. Therefore, clinical stage 1s was assumed, and the patient underwent chemotherapy with three courses of BEP-chemotherapy with no intercurrent complications. Follow-up is uneventful, with no recurrence five years after completion of treatment. Ultrasound of the left testis did not detect any pathologies, therefore no radiation of the left testis was initiated. The patient also showed mild neuromuscular symptoms with observed contracture of the arms. However, due to intensive specific physical exercise, the patient is not impacted by this symptom in daily life.

Investigation of the present family (37 family members) by Knoblauch et al. revealed a total of nine male family members carrying the X-chromosomal FHL-1 mutation [6]. To date, no other family member has been diagnosed with GCT.

## Discussion

A familial occurrence of testicular GCT is observed in 1-2% of all cases with GCT [7]. Brothers and sons/fathers of GCT patients have an eight to 10 and four to six-fold increased risk of GCT, respectively, and the risk is even higher in twin brothers [8]. Genetic analysis of GCT tissue revealed genomic alterations, including gains in chromosome 12p, and mutations in KIT, KRAS, and NRAS, particularly in patients with seminomas. Recent investigations have identified chromosome Y gr/gr deletion, and mutations in the PDE11A gene, as genetic modifiers for increased GCT risk [9]. Furthermore, three additional susceptibility loci, KITLG, SPRY4, and BAK1 have been identified. To date, more than 30 genome risk loci for GCT were identified; however, no single gene could be identified that would explain the familial occurrence of testicular GCT. Cumulative evidence suggests that inheritance is likely driven by a complex polygenic model [10]. None of these mutations is linked with EDMD.

Familial testicular GCT is clinically characterized by younger age at presentation, histological concordance of testicular neoplasms, and a high prevalence of bilateral disease [11]. Accordingly, the clinical features of the two patients reported here are consistent with that experience since both patients were younger than the average testis cancer patient, the two neoplasms were of concordant histology, and one of the brothers had contralateral germ cell neoplasia in situ, additionally.

EDMD is a rare muscular dystrophy, its incidence is 0.39 in 100,000 individuals per year [12]. Clinically, EDMD is characterized by the triad of (1) joint contractures that come into being in early childhood, (2) slowly progressive muscle weakness, and wasting initially in a humeroperoneal distribution, and later extending to the scapular and pelvic girdle muscles, and (3) cardiac involvement [13].

Windpassinger et al. and Schwartzmeier et al. reported patients are generally athletic [14,15]. However, meticulous physical examination revealed weakness and atrophy of postural muscles, whereas other muscles appeared hypertrophic. Furthermore, EDMD patients frequently develop significant contractures of tendons of the lower limb, have a short neck, and have limited neck flexion as well as extension. Other common clinical features involve scoliosis, back pain, and gait problems. The second clinical hallmark of EDMD involves cardiac abnormalities, particularly, arrhythmias and cardiomyopathy. Skeletal muscle symptoms usually precede cardiac involvement, and the disorder is progressive in all patients [14,15]. Blood analysis usually revealed increased serum creatine kinase levels. Clinical diagnosis is based on the typical clinical features and skeletal muscle biopsy, which discloses myopathic or dystrophic changes as well as molecular genetic testing [13].

The later course may involve pneumothorax formation and finally respiratory failure secondary to severe scoliosis. The primary cause of death in patients with EDMD is life-threatening cardiac conduction blocks and heart failure [16].

EDMD is not a homogeneous clinical entity because it is associated with various gene mutations, all of which trigger slightly different clinical EDMD types. Both X-linked and autosomal forms of EDMD occur, with the X-linked form being described first in 1966 by Emery and Dreifuss [17]. One common mutation relates to the gene of FHL-1, also referred to as skeletal muscle LIM-protein 1 (SLIM-1). FHL-1 belongs to the LIM protein family in humans, which is highly expressed in skeletal and cardiac muscle. The FHL-1 gene is located on the X chromosome (Xq26.3.) and, as suggested by the name, contains four and a half LIM domains [6,18-19]. The physiological role of FHL-1 is to regulate gene transcription, cytoarchitecture, cell proliferation, and signal transduction. The first FHL-1 gene mutation was reported in 2008 by Cowling and Cottle [20]. The pathogenetic pathway related to the clinical features of EDMD is still unclear.

Several genes have been implicated in the pathogenesis of EDMD. So far, only 35% of EDMD cases are genetically elucidated and associated with EMD or LMNA gene mutations (the two most common genes) [12]. Other genes that have been associated with this disease are listed in Table 1. There are still more causative genes yet to be discovered.

**Table 1:** Overview of the genetics of EDMD Source: [12] EDMD = Emery-Dreifuss muscular dystrophy; EMD = Emery-Dreifuss dystrophy; FHL-1 = Four and a half Limb domain protein 1; LMNA = Lamin A; TMEM43 = transmembrane protein 43

Gene	Gene product	Locus	Inheritance	Typ of EDMD	Major Symptoms
EMD	Emerin	Xq28	X-linked recessive	EDMD 1	Contractures, weakness of humeroperoneal muscles, cardiomyopathy
LMNA	Lamin A/C	1q22	Autosomal dominant	EDMD 2	Especially dilative cardiomyopathy
LMNA	Lamin A/C	1q22	Autosomal recessive	EDMD 3	Especially dilative cardiomyopathy
FHL-1	FHL 1	Xq26.3	X-linked recessive	EDMD 6	Especially muscle atrophy in the pelvic, peroneal, and scapular regions
TMEM43	LUMA	3p.25.1	Autosomal dominant	EDMD 7	Usually slowly progressive muscle weakness and atrophy of the proximal muscles

Of note, no malignant disease has been found to be associated with EDMD to date, thus the present cases are unprecedented, so far.

## Conclusions

EDMD is a genetically inherited disease predominantly involving the musculoskeletal system and, secondly, the cardiovascular system. To date, there have been no reported cases of the co-occurrence of EDMD and testicular disease. Therefore, the biological link between GCT and EDMD remains elusive and the association of EDMD with familial testicular tumors as observed in our patients can probably not be explained by the biological characteristics of the inherited disease. Thus, the simultaneous occurrence of both testicular cancer and EDMD in a pair of brothers appears to be a chance event. However, we cannot entirely exclude any unrecognized genetic interrelationships between the two diseases, and we thus wish to document this extraordinary coincidence of diseases for future research.
